# Perinatal mortality rate and adverse perinatal outcomes presumably attributable to placental dysfunction in (near) term gestation: A nationwide 5-year cohort study

**DOI:** 10.1371/journal.pone.0285096

**Published:** 2023-05-04

**Authors:** Stefanie Elisabeth Damhuis, Hester Dorien Kamphof, Anita C. J. Ravelli, Sanne Jehanne Gordijn, Wessel J. Ganzevoort

**Affiliations:** 1 Department of Obstetrics and Gynecology, Amsterdam University Medical Centers, University of Amsterdam, Amsterdam, The Netherlands; 2 Department of Obstetrics and Gynecology, University Medical Center Groningen, University of Groningen, Groningen, The Netherlands; 3 Amsterdam Reproduction and Development Research Institute, University of Amsterdam, Amsterdam, The Netherlands; 4 Department of Medical Informatics, Amsterdam University Medical Centers, University of Amsterdam, Amsterdam, The Netherlands; University Medical Centre Ljubljana (UMCL) / Faculty of Medicine, University Ljubljana (FM,UL), SLOVENIA

## Abstract

**Introduction:**

Placental dysfunction can lead to perinatal hypoxic events including stillbirth. Unless there is overt severe fetal growth restriction, placental dysfunction is frequently not identified in (near) term pregnancy, particularly because fetal size is not necessarily small. This study aimed to evaluate, among (near) term births, the burden of hypoxia-related adverse perinatal outcomes reflected in an association with birth weight centiles as a proxy for placental function.

**Material and method:**

A nationwide 5-year cohort of the Dutch national birth registry (PeriNed) including 684,938 singleton pregnancies between 36^+0^ and 41^+6^ weeks of gestation. Diabetes, congenital anomalies, chromosomal abnormalities and non-cephalic presentations at delivery were excluded. The main outcome was antenatal mortality rate according to birthweight centiles and gestational age. Secondary outcomes included perinatal hypoxia-related outcomes, including perinatal death and neonatal morbidity, analyzed according to birthweight centiles.

**Results:**

Between 2015 and 2019, 1,074 perinatal deaths (0.16%) occurred in the study population (n = 684,938), of which 727 (0.10%) antenatally. Of all antenatal- and perinatal deaths, 29.4% and 27.9% occurred in birthweights below the 10^th^ centile. The incidence of perinatal hypoxia-related outcomes was highest in fetuses with lowest birthweight centiles (18.0%), falling gradually up to the 50^th^ and 90^th^ centile where the lowest rates of hypoxia-related outcomes (5.4%) were observed.

**Conclusion:**

Perinatal hypoxia-related events have the highest incidence in the lowest birthweight centiles but are identifiable throughout the entire spectrum. In fact, the majority of the adverse outcome burden in absolute numbers occurs in the group with a birthweight above the 10^th^ centile. We hypothesize that in most cases these events are attributable to reduced placental function. Additional diagnostic modalities that indicate placental dysfunction at (near) term gestation throughout all birth weight centiles are eagerly wanted.

## Introduction

Stillbirth at term gestation is one of the most feared complications of pregnancy and affects every practicing obstetrician. Sometimes, a clear and unavoidable cause can be identified, such as the occurrence of congenital anomalies, placental abruption or a cord prolapse. However, in the majority of cases the cause remains unknown [1, 2]. Under the assumption that most stillbirths are preceded by a period of hypoxia, the placenta likely plays a prominent role in the majority of unexplained deaths.

Placental dysfunction is the most common underlying pathophysiologic mechanism of fetal growth restriction (FGR), defined as a fetus that does not reach its intrinsic growth potential [3–5]. Small for gestational age (SGA), defined as an estimated fetal weight or birthweight below the 10^th^ percentile, is often used as a proxy for FGR. However, fetuses can be constitutionally small but healthy whereas FGR is essentially a pathological condition in which the fetus is not necessarily small [4].

In placental dysfunction there is a mismatch between the placental metabolic- and gaseous exchange and fetal demands [6, 7]. Metabolic exchange failure leads to inappropriate fetal growth and development. Gaseous exchange failure leads to hypoxia, acute and chronic, starting from early in pregnancy up to during delivery. In late-onset placental dysfunction, gaseous exchange failure may occur before the metabolic exchange failure becomes apparent through suboptimal fetal growth. If the ensuing hypoxia goes without timely intervention, the fetus is at increased risk for adverse perinatal outcomes [8, 9], ranging from stillbirth to emergency delivery for fetal compromise, short-term neonatal morbidity and long-term impact on neurodevelopment, including hypoxic ischemic encephalopathy (HIE) and cerebral palsy [6, 10–14].

Assessment of fetal size, preferably sequential, together with other functional placental markers (such as Doppler ultrasound and maternal biomarkers) are cornerstones of prenatal care to identify fetuses suffering from reduced placental function. For clinical and research purposes, the ‘fetus-at-risk’ has traditionally been defined by small size, usually below the 10^th^ centile (SGA) [15]. Most studies analyze dichotomized comparisons between groups of SGA and appropriate for gestational age (AGA) fetuses and newborns, consistently identifying SGA as a risk factor for adverse outcomes [16]. Only few studies have focused on the association between fetal and/or newborn size for gestational age (as a function of reduced placental function) and adverse outcomes with other birth weight centiles thresholds or in a semi-continuous measure, describing the association across the whole spectrum of growth centiles [17–21]. For this reason, and in the absence of systematic evaluations of placental function in AGA fetuses, accurate estimates of the burden of hypoxia-related adverse outcomes are lacking among AGA fetuses. This is particularly important in late gestation when reduced placental function is not restricted to small fetal size [7, 22, 23].

The aim of this nationwide cohort study was to evaluate, among (near) term births, the burden of hypoxia-related adverse perinatal outcomes reflected in an association with birthweight centiles as a proxy for placental function.

## Material and methods

This population cohort study was conducted with the data from the national perinatal registry (PeriNed) in the Netherlands (www.perined.nl) [24]. Participation in the registry is obligatory for midwife practices, obstetricians and neonatologists, and 96–98% of births are recorded in the registry [25]. The data include anonymized information about pregnancy, childbirth and hospital (re)admissions of newborns. Data about Doppler measurements, maternal BMI, smoking, maternal biomarkers and placenta histology are not included in the registry. The data is recorded at the discretion of the caregiver and is annually checked for consistency.

We included all (near) term singletons born between 36^+0^ and 41^+6^ weeks of gestation from 1^st^, 2015 to December 31^st^, 2019. We excluded pregnancies complicated by congenital anomalies, chromosomal abnormalities, non-cephalic presentation at time of birth and pregnancies complicated with any type of diabetes.

The primary outcomes were antenatal- and perinatal death rates. Perinatal death was defined as antenatal deaths, intrapartum deaths and neonatal deaths within 7 days after birth. They were analyzed according to birthweight centiles and gestational age, and separately in the subgroup SGA (defined as a birthweight <10^th^ centile according to Hoftiezer reference charts). [26] Antenatal death risks were calculated for each birth weight percentile group at 36, 37, 38, 39, 40 and 41 weeks of gestation using a ‘‘fetus-at-risk” approach, dividing the number of stillbirths in that gestational age period by the number of women at risk and expressed as the average weekly risk [27].

Secondary outcomes were divided into three predetermined outcome groups that are deemed to be related to perinatal hypoxia as a result of reduced placental function. ‘‘Outcome-1” was defined as severe adverse outcomes of pregnancy including: perinatal mortality (antenatal-, perinatal- or neonatal death <28 days) or hypoxic ischemic encephalopathy (HIE). “Outcome-2” was defined as adverse labor outcomes, including Apgar <7 and/or neonatal intensive care (NICU) admission >24 hours and/or emergency delivery for (presumed) fetal compromise. Fetal compromise was user-defined, as reported by the caregiver. Fetal compromise in combination with obstructed labor as indication for an emergency delivery was included, whereas obstructed labor as sole indication was excluded. ‘‘Outcome-3” was defined as adverse neonatal outcomes including necrotizing enterocolitis (NEC) and/or neonatal hypoglycemia and/or neonatal hypothermia and/or respiratory distress syndrome (RDS) and/or bronchopulmonary dysplasia (BPD) and/or neonatal convulsions.

Secondary outcomes were analyzed according to birthweight centiles, calculated from Hoftiezer population reference charts [26].

### Statistical analysis

Data analyses were performed with SAS version 9.4 Baseline characteristics and outcome parameters were described using descriptive statistics. Categorical variables were presented as proportions (%) and continuous variables as means (± standard deviations (SDs)) or medians (interquartile range). Antenatal- and perinatal death, and adverse outcome rates were tested by gestational age with a chi-square test. Rates for all adverse outcomes (‘‘outcome 1–3”) were tested by birthweight centile groups with a chi-square test. A p-value of <0.001 was considered significant.

There were five missing Apgar scores and 1058 missing values for birthweight centiles coded as unknown. The missing data (18 maternal age and 816 birthweights) were imputed with single imputation.

### Ethical approval

No ethical approval was needed according to the Dutch central committee of Human Research, as the study only used previously collected cohort data. All data in this study were fully anonymized before we accessed them. Ethical approval was obtained by the committee for research and ethics of the PeriNed registry (approval number 19.41).

### Patient involvement

Because this was a nationwide pseudonymised register-based study of the Netherlands, neither participants nor the public were involved in developing the research question or in the design, management, or interpretation of the study. We adhered as much as possible to the core outcome set for fetal growth restriction that has been developed with the involvement of patients [28].

## Results

The PeriNed database contained 764,529 singleton pregnancies with the time of birth between 36^+0^ and 41^+6^ weeks of gestation between January 1^st^, 2015, and December 31^st^, 2019. We excluded 79,591 cases because of diabetes, chromosomal abnormalities, congenital anomalies, or non-cephalic presentation at the time of birth, leaving 684,938 births for the current study. Characteristics of the study population and neonatal outcomes are shown in Table 1.

**Table 1:** Maternal and neonatal baseline characteristics and outcomes by gestational week.

Maternal	Gestational age (weeks)					
	36 *n = 14*,*049*	37 *n = 45*,*031*	38 *n = 103*,*672*	39 *n = 182*,*770*	40 *n = 212*,*415*	41 *n = 127*,*001*
Maternal age, mean, (SD)	30.4 (4.9)	30.5 (4.9)	30.7 (4.9)	30.7 (4.7)	30.6 (4.6)	30.8 (4.6)
Nulliparous	53.6%	46.0%	39.7%	39.3%	42.5%	49.2%
Caucasian ethnicity	89.8%	89.1%	88.2%	87.9%	89.6%	90.9%
Hypertensive pregnancy disorder	11.5%	15.9%	9.3%	5.1%	3.9%	3.1%
Previous caesarean section	6.9%	7.6%	11.4%	11.6%	5.2%	5.0%
Onset of labor						
*Spontaneous*	70.2%	50.8%	54.6%	72.7%	86.6%	68.8%
*Labor induction*	18.3%	39.3%	32.6%	15.8%	11.3%	28.7%
*Planned caesarean section*	8.6%	6.8%	10.3%	9.9%	0.9%	0.9%
*Unknown*	2.9%	3.0%	2.4%	1.6%	1.2%	1.7%
Mode of delivery						
*Spontaneous*	74.4%	77.9%	75.4%	76.9%	82.8%	75.9%
*Instrumental vaginal delivery*	7.6%	6.4%	6.2%	6.3%	8.1%	10.5%
*Emergency caesarean section*	7.0%	6.9%	6.2%	5.0%	6.5%	10.7%
*Planned caesarean section*	8.6%	6.8%	10.3%	9.9%	0.9%	0.9%
*Unknown*	2.4%	2.0%	1.9%	1.9%	1.9%	2.0%
Meconium-stained liquor	3.3%	3.1%	5.3%	11.0%	18.3%	22.2%
**Neonatal**						
Male sex	55.5%	53.0%	55.4%	51.6%	49.9%	50.8%
Birthweight (grams), mean, (SD)	2789 (430)	3011 (447)	3284 (442)	3451 (418)	3606 (417)	3731 (429)
Hoftiezer centile, mean, (SD)	46.3 (29.9)	47.5 (30.2)	51.4 (29.9)	50.5 (28.9)	50.0 (28.4)	50.1 (28.9)
SGA (<p10)	15.6%	14.7%	10.9%	9.9%	9.5%	9.4%
LGA (>p90)	9.4%	10.5%	13.2%	11.1%	10.0%	9.9%
Apgar 5-minutes <7	2.6%	1.5%	1.1%	0.9%	1.1%	1.6%
Apgar 5-minutes <4	0.4%	0.2%	0.1%	0.1%	0.1%	0.2%
NICU admission >24 hours	3.0%	1.4%	0.7%	0.4%	0.5%	0.6%
Cord pH determined	18.1%	10.0%	7.9%	7.1%	6.3%	6.9%
*pH <7*.*1 (if determined)*	1.7%	0.9%	0.8%	0.7%	0.9%	1.3%

SD: standard deviation

SGA: small for gestational age (birthweight <p10)

LGA: large for gestational age (birthweight >p90)

NICU: neonatal intensive care admission

### Mortality rates

In this five-year period, 727 antenatal deaths (0.10%), 1,074 perinatal deaths within 7 days (0.16%) and 1,119 (0.16%) perinatal deaths within 28 days occurred in our study population (Table 2). Of all antenatal- and perinatal deaths, 29.4% and 27.9% occurred in SGA fetuses and newborns. Gestational age had a significant association with antenatal- and perinatal death rate (p<0.0001).

**Table 2:** Antenatal and perinatal death rates according to gestational age in weeks of 684,938 singleton pregnancies in the Netherlands from 2015 to 2019.

Gestational age (weeks)	Pregnancies	Antenatal death			Perinatal death		
	n	n	%	SGA	n	%	SGA
36	14,049	123	0.88%	39.0%	149	1.06%	38.3%
37	45,031	122	0.27%	25.4%	150	0.33%	24.0%
38	103,672	137	0.13%	24.1%	200	0.19%	22.0%
39	182,770	141	0.08%	30.5%	220	0.12%	30.9%
40	212,415	120	0.06%	26.7%	201	0.09%	26.9%
41	127,001	84	0.07%	32.0%	154	0.12%	26.6%
**Total**	**100% (n = 684,938)**		**0.10% (n = 727)**	**29.4% (n = 214)**		**0.16% (n = 1074)**	**27.9% (n = 300)**

SGA: small for gestational age (birthweight <p10)

Perinatal death includes antenatal-, intrapartum and neonatal death within 7 days postpartum.

**Fig 1:**
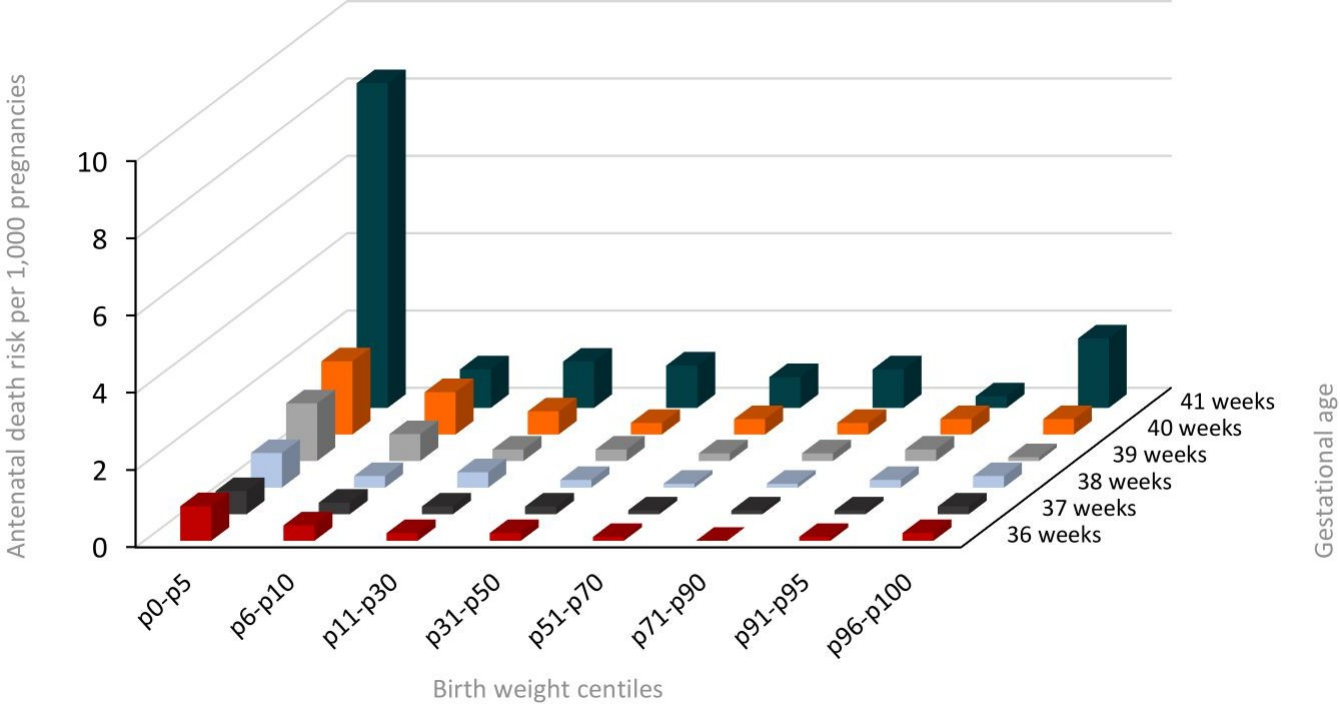
Average antenatal death risk per 1,000 pregnancies for each birth weight centile group at gestational age week 36, 37, 38, 39, 40 and 41 in the Netherlands from 2015 to 2019.

The weekly prospective risk of antenatal death increased with advanced gestational age and was highest in the lowest birth weight centiles. The prospective stillbirth risk decreased strongly with increasing birth weight percentiles up to the p70-p90 at all gestational weeks, except for week 41 (Fig 1 and S1 Table in S1 File).

### Adverse events

**Fig 2:**
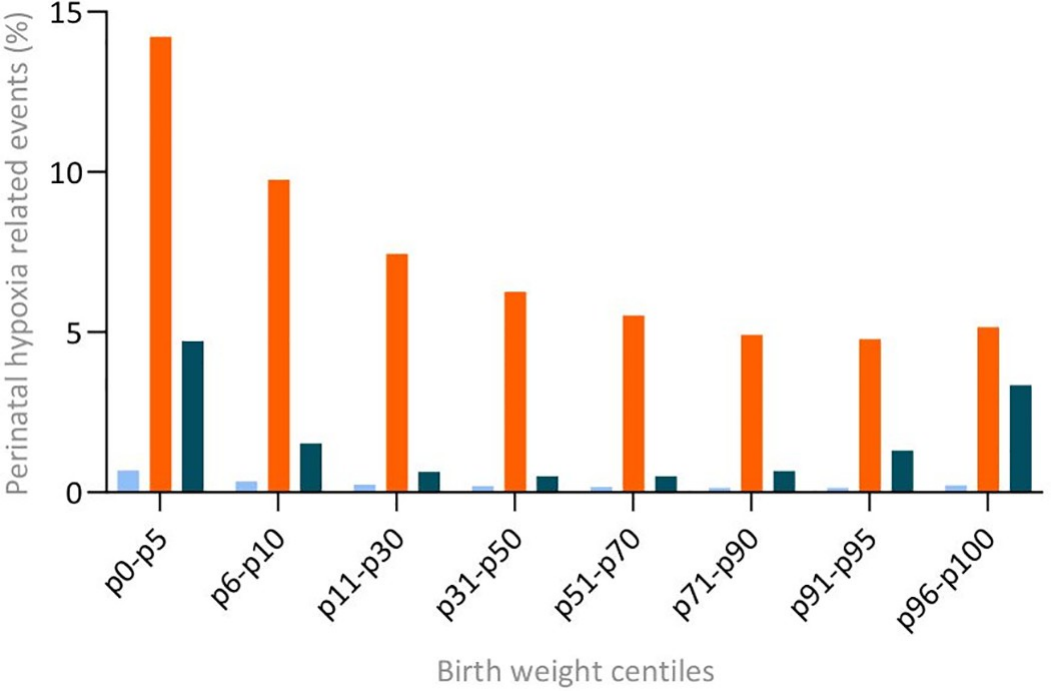
Perinatal hypoxia related events according to birth weight centile groups for neonates born between 36^+0^ and 41^+6^ weeks’ gestation in the Netherlands from 2015 to 2019. Light blue square Outcome 1—Severe adverse outcomes of pregnancy: perinatal mortality or HIE (including antenatal death, perinatal death and neonatal death within 28 days). orange square Outcome 2—Adverse labor outcomes: Apgar <7 and/or NICU admission (>24 hrs) and/or emergency delivery for fetal compromise. dark blue square Outcome 3: Adverse neonatal outcomes including any of the following: necrotizing enterocolitis, neonatal hypoglycemia, neonatal hypothermia, respiratory distress syndrome, bronchopulmonary dysplasia, and neonatal convulsions.

A total of 53,272 (7.8%) adverse events were described. Severe adverse outcomes of pregnancy (“outcome-1”) was observed in 0.2%, adverse labor outcomes (“outcome-2”) was observed in 6.5%, and adverse neonatal outcomes (“outcome-3”) occurred in 1.0% (Table 3). Highest absolute numbers were found at 36 weeks of gestation and lowest rates at 39–40 weeks of gestation (P<0.001). The distribution of the three outcomes according to birthweight centiles is shown in Fig 2. Percentages of perinatal hypoxia related events according to birth weight centile groups can be found in the Appendix, S2 Table in S1 File. The incidence of each outcome was highest in fetuses with the lowest birthweight centiles, falling gradually up to the p50-p90 at which the lowest rates of adverse hypoxia-related outcomes occurred.

**Table 3:** Perinatal hypoxia related events according to gestational age for singleton pregnancies in the Netherlands from 2015 to 2019.

Gestational age (weeks)		Outcome 1 *Severe adverse pregnancy outcome*		Outcome 2 *Adverse labor outcome*		Outcome 3 *Adverse neonatal outcome*	
	n	n	%	n	%	n	%
36	14,049	168	1.20	1,531	10.90	1,175	8.36
37	45,031	184	0.41	3,348	7.43	1,245	2.76
38	103,672	251	0.24	5,741	5.54	1,351	1.30
39	182,770	300	0.16	9,147	5.00	1,170	0.64
40	212,415	329	0.15	13,431	6.32	1,207	0.57
41	127,001	280	0.22	11,618	9.15	796	0.63
**Total**	**684,938**	**1,512**	**0.22**	**44,816**	**6.54**	**6,944**	**1.01**

Outcome 1—Severe adverse outcomes of pregnancy: perinatal mortality or HIE (including antenatal death, perinatal death and neonatal death within 28 days).

Outcome 2—Adverse labor outcomes: Apgar <7 and/or NICU admission (>24 hrs) and/or emergency delivery for fetal compromise.

Outcome 3—Adverse neonatal outcomes including any of the following: necrotizing enterocolitis, neonatal hypoglycemia, neonatal hypothermia, respiratory distress syndrome, bronchopulmonary dysplasia, and neonatal convulsions.

## Discussion

### Main findings

This study assessed the perinatal hypoxia-related events, including perinatal mortality, throughout the birthweight-for-gestation spectrum in singleton pregnancies between 36^+0^ and 41^+6^ weeks of gestation within a nationwide Dutch cohort. Our study shows that these adverse perinatal events, have the highest concentration in lowest birthweight centiles. However, they are identifiable throughout the entire spectrum, and in absolute numbers they are most frequent in appropriate for gestational age infants.

The gradual association between birthweight centiles and adverse outcomes was observed in all three composite outcome groups, with the lowest burden between the 50^th^ and 90^th^ centile. Remarkably this pattern was less obvious to the adverse neonatal morbidity rates (‘Outcome 3’). Although a high incidence of events was reported in newborns with a birthweight below the 10^th^ percentile and above the 90^th^ percentile, there was no gradual drop of incidence rates between the 10^th^ and 90^th^ centile. We hypothesize that protocolized monitoring and treatment of the newborn focuses on SGA and large for gestational age (LGA), and that the observed high rates of hypoglycemia are the result of affirmation bias due to targeted testing. Hypoglycemia incidence rates in AGA are largely unknown, and a study that reported glucose levels in term newborns of appropriate size excluded infants suspected of intrapartum hypoxia [29].

It is known that fetal overgrowth is associated with a threefold higher risk for stillbirth independent of maternal diabetic status and is a risk factor for neonatal death and morbidity [30, 31]. Mortality- and morbidity in the birthweights above the 90^th^ centile are typically not attributed to growth restriction or the underling mechanism of placental dysfunction. The lack of a gold standard of placental insufficiency hinders firm conclusions. However, it can validly be reasoned that the pattern of the rising incidence of hypoxia-related event rates is most likely a manifestation of (acute) failure of the gaseous exchange of the placenta. This may present without obvious declined growth from chronic metabolic exchange failure because placental reserve in term pregnancy is already limited. For that reason, fetuses with a high genetic growth potential do not have the time to ‘travel’ to the lowest percentiles before they suffer from the risks of acidosis. Undetected or unreported diabetes and other underlying placenta pathology such as terminal villous deficiency may also mediate this process [32].

Although we excluded emergency deliveries for the indication of obstructed labor from analysis, obstructed labor combined with fetal compromise as indication for emergency delivery was included as well as neonatal hypoglycemia without further distinction. This may have also contributed to the rise in morbidity and mortality rates in our analyses.

Some of the observed patterns are skewed due to the interventions starting delivery indicated by clinical findings such as signs of FGR/SGA, hypertensive disorders of pregnancy and suspected macrosomia. Since antenatal death is prevented by early delivery, these biases likely have a bearing on the risks of all outcomes. This does not negate the observation that the majority of fetal deaths occurs within the ‘normal’ birthweight centile groups, of which a significant proportion have not been diagnosed as ‘at risk’ And may not have received the appropriate interventions.

Quite a substantial proportion of the adverse outcomes that we found in this dataset were ‘emergency deliveries for fetal compromise’. Fetal compromise is usually suspected if the cardiotocography (CTG) becomes abnormal. However, the diagnostic accuracy of the CTG is low and if continuous intrapartum CTG monitoring is installed this increases the rate of emergency instrumental deliveries and caesarean sections [33]. Furthermore, it is possible that clinicians aware of a fetus diagnosed with FGR are more likely to report fetal compromise, which potentially can over-emphasize fetal compromise at lower birth weight centiles. Presumably our dataset contains a number of false positives from fetal compromise. However, we do not expect that this has had a noteworthy influence on the trend that we have shown.

### Interpretation

We hypothesize that the majority of the hypoxia-related events are presumably attributable to placental dysfunction, also in the appropriate for gestational age spectrum.

Placental insufficiency after birth can be diagnosed through pathological analyses of the placenta and/or perinatal autopsy. However, in late-onset FGR placental function can deteriorate rapidly and obvious signs such as accelerated parenchymal maturation, nucleated red blood cells and reduced liver to brain ratio might be absent. Limited placental reserve at term can be seen as a ‘common’ phenomenon as exemplified by the universal flattening of the fetal growth curve in late gestation. This is strengthened by current study in which an increasing stillbirth risk beyond 41- and 42 weeks of gestation was observed. We suspect that this contributes to the fact that although pathological analyses of the placenta are performed, still 30–60% of stillbirth cases remain unexplained, and that the same lesions are seen in placentas of ‘normal’ pregnancies [1, 2, 34].

The order of magnitude of the issue is that in the context of a high-income country, we observed that one out of 1,000 singleton non-anomalous pregnancies without diabetes ends in antenatal death at (near) term. We postulate that most of these deaths most likely result from placental dysfunction. In this population, over 70% of antenatal and perinatal deaths occurred in fetuses and newborns that were not SGA and apparently appropriately grown. A considerable proportion of these deaths could potentially be prevented if timely identification of the compromised fetus suffering from placental dysfunction and installation of adequate monitoring and interventions would become possible. This means that, for example in the United Kingdom, about 400 antenatal deaths in the (near) term period might be prevented annually. It underlines the pressing need for proven evaluated diagnostic strategies, since the contemporary clinical diagnostic strategy with the focus on fetal size (and screening for SGA) is inaccurate [35].

We excluded multiple pregnancies, congenital anomalies, chromosomal abnormalities, maternal (gestational) diabetes and non-cephalic presentation at the time of birth as they are known causes and risk factors of fetal death. Karyotyping and autopsy is advised in case of perinatal death in the Netherlands and results are reported in the registry. Although we acknowledge that the causes of death in this dataset are miscellaneous and not necessarily related to reduced placental function, we postulate that most other causes of death (like cord accidents, placental abruption, and uterine rupture) are rare events in (near) term births. For instance, the risk for placental abruption during pregnancy is 0.22% (and this is lower in term pregnancies: 0.08% for pregnancies between 39–41 weeks), and the risk for uterine rupture in women with a previous caesarean section is 0.20% [36, 37]. It is known that 30–60% of stillbirths remains unexplained. In particular these unexplained cases are frequently associated with placental dysfunction [38, 39]. Therefore we consider that placental dysfunction is a prominent mechanism in most perinatal death cases in this dataset. The gradual association of all hypoxia-related events with birthweight centiles lends further support to the hypothesis that the majority of deaths can be attributed to placental dysfunction.

### Strengths and limitations

A strength of this study is that the dataset of the perinatal registry is comprehensively based on nearly all births in the Netherlands that occurred between 2015 and 2019. A limitation is however that data on maternal smoking and BMI, both possible confounders, are absent in the registry. Also, the perinatal registry lacks detailed information on causes of perinatal deaths, and on additional supportive information pointing towards reduced placental function including Doppler measurements, maternal biomarkers and placental histopathology reports.

## Conclusion

Perinatal hypoxia-related events have the highest concentration in lowest birthweight centiles, but are identifiable throughout the entire spectrum. In fact, the majority of the adverse perinatal outcome burden in absolute numbers occurs above the 10^th^ birthweight centile. We hypothesize that in most cases these events are attributable to reduced placental function. Evaluation of additional diagnostic modalities that indicate placental dysfunction at (near) term gestation throughout all birthweight centiles is eagerly wanted. Meanwhile, the current study is a good indicator for clinical obstetric practice to recognize that hypoxia related events affect both SGA and AGA fetuses.

## Supporting information

S1 File (DOCX)

## Abbreviations

AGA: Appropriate for gestational age
BPD: Bronchopulmonary dysplasia
CTG: Cardiotocography
FGR: Fetal growth restriction
HIE: Hypoxic ischemic encephalopathy
NEC: Necrotizing enterocolitis
RDS: Respiratory distress syndrome
SD: Standard deviation
SGA: Small for gestational age

## References

[1] Hoyert DL, Gregory EC. Cause of fetal death: data from the fetal death report, 2014. 2016. 27805550

[2] Man J, Hutchinson JC, Heazell aE, Ashworth M, Levine S, Sebire NJ. Stillbirth and intrauterine fetal death: factors affecting determination of cause of death at autopsy. Ultrasound in Obstetrics and Gynecology. 2016;48(5):566–73. doi: 10.1002/uog.16016 27781317

[3] Burton GJ, Jauniaux E. Pathophysiology of placental-derived fetal growth restriction. American Journal of Obstetrics and Gynecology. 2018;218(2):S745–S61. 29422210 10.1016/j.ajog.2017.11.577

[4] Gordijn S, Beune I, Thilaganathan B, Papageorghiou A, Baschat A, Baker P, et al. Consensus definition of fetal growth restriction: a Delphi procedure. Ultrasound in Obstetrics & Gynecology. 2016;48(3):333–9. doi: 10.1002/uog.15884 26909664

[5] Mifsud W, Sebire NJ. Placental pathology in early-onset and late-onset fetal growth restriction. Fetal diagnosis and therapy. 2014;36(2):117–28. doi: 10.1159/000359969 24577279

[6] Turner JM, Mitchell MD, Kumar SS. The physiology of intrapartum fetal compromise at term. American journal of obstetrics and gynecology. 2020;222(1):17–26. doi: 10.1016/j.ajog.2019.07.032 31351061

[7] Coutinho CM, Melchiorre K, Thilaganathan B. Stillbirth at term: Does size really matter? International Journal of Gynecology & Obstetrics. 2020;150(3):299–305. doi: 10.1002/ijgo.13229 32438457

[8] Barker DJ. Adult consequences of fetal growth restriction. Clinical obstetrics and gynecology. 2006;49(2):270–83. doi: 10.1097/00003081-200606000-00009 16721106

[9] Miller SL, Huppi PS, Mallard C. The consequences of fetal growth restriction on brain structure and neurodevelopmental outcome. The Journal of physiology. 2016;594(4):807–23. doi: 10.1113/JP271402 26607046 PMC4753264

[10] Burton GJ, Fowden AL, Thornburg KL. Placental origins of chronic disease. Physiological reviews. 2016;96(4):1509–65. doi: 10.1152/physrev.00029.2015 27604528 PMC5504455

[11] Walker D, Marlow N. Neurocognitive outcome following fetal growth restriction. Archives of Disease in Childhood-Fetal and Neonatal Edition. 2008;93(4):F322–F5. doi: 10.1136/adc.2007.120485 18381841

[12] Glass HC, Glidden D, Jeremy RJ, Barkovich AJ, Ferriero DM, Miller SP. Clinical neonatal seizures are independently associated with outcome in infants at risk for hypoxic-ischemic brain injury. The Journal of pediatrics. 2009;155(3):318–23. doi: 10.1016/j.jpeds.2009.03.040 19540512 PMC3014109

[13] Ronen GM, Penney S, Andrews W. The epidemiology of clinical neonatal seizures in Newfoundland: a population-based study. The Journal of pediatrics. 1999;134(1):71–5. doi: 10.1016/s0022-3476(99)70374-4 9880452

[14] Vasudevan C, Levene M, editors. Epidemiology and aetiology of neonatal seizures. Seminars in Fetal and Neonatal Medicine; 2013: Elsevier. 10.1016/j.siny.2013.05.008 23746578

[15] Gordijn SJ, Beune IM, Ganzevoort W. Building consensus and standards in fetal growth restriction studies. Best Pract Res Clin Obstet Gynaecol. 2018;49:117–26. doi: 10.1016/j.bpobgyn.2018.02.002 29576470

[16] Sacchi C, Marino C, Nosarti C, Vieno A, Visentin S, Simonelli A. Association of intrauterine growth restriction and small for gestational age status with childhood cognitive outcomes: a systematic review and meta-analysis. JAMA pediatrics. 2020;174(8):772–81. doi: 10.1001/jamapediatrics.2020.1097 32453414 PMC7251506

[17] Vasak B, Koenen S, Koster M, Hukkelhoven C, Franx A, Hanson M, et al. Human fetal growth is constrained below optimal for perinatal survival. Ultrasound in Obstetrics & Gynecology. 2015;45(2):162–7. doi: 10.1002/uog.14644 25092251

[18] Kamphof HD, Gordijn SJ, Ganzevoort W, Verfaille V, Offerhaus PM, Franx A, et al. Associations of severe adverse perinatal outcomes among continuous birth weight percentiles on different birth weight charts: a secondary analysis of a cluster randomized trial. BMC Pregnancy and Childbirth. 2022;22(1):1–11. 35490210 10.1186/s12884-022-04680-5 PMC9055757

[19] Iliodromiti S, Mackay DF, Smith GC, Pell JP, Sattar N, Lawlor DA, et al. Customised and noncustomised birth weight centiles and prediction of stillbirth and infant mortality and morbidity: a cohort study of 979,912 term singleton pregnancies in Scotland. PLoS medicine. 2017;14(1):e1002228. doi: 10.1371/journal.pmed.1002228 28141865 PMC5283655

[20] Vieira MC, Relph S, Persson M, Seed PT, Pasupathy D. Determination of birth-weight centile thresholds associated with adverse perinatal outcomes using population, customised, and Intergrowth charts: A Swedish population-based cohort study. PLoS Medicine. 2019;16(9):e1002902. doi: 10.1371/journal.pmed.1002902 31539391 PMC6754137

[21] Francis JH, Permezel M, Davey MA. Perinatal mortality by birthweight centile. Australian and New Zealand Journal of Obstetrics and Gynaecology. 2014;54(4):354–9. doi: 10.1111/ajo.12205 24731210

[22] Damhuis SE, Ganzevoort W, Gordijn SJ. Abnormal Fetal Growth: Small for Gestational Age, Fetal Growth Restriction, Large for Gestational Age: Definitions and Epidemiology. Obstetrics and Gynecology Clinics. 2021;48(2):267–79. doi: 10.1016/j.ogc.2021.02.002 33972065

[23] Bardien N, Whitehead CL, Tong S, Ugoni A, McDonald S, Walker SP. Placental insufficiency in fetuses that slow in growth but are born appropriate for gestational age: a prospective longitudinal study. PLoS One. 2016;11(1):e0142788. doi: 10.1371/journal.pone.0142788 26730589 PMC4701438

[24] Perined, Perinatal Care in the Netherlands. Utrecht2015-2019.

[25] Perined, Perinatale zorg in Nederland anno 2019: landelijke perinatale cijfers en duiding. Utrecht2020.

[26] Hoftiezer L, Hof MH, Dijs-Elsinga J, Hogeveen M, Hukkelhoven CW, Van Lingen RA. From population reference to national standard: new and improved birthweight charts. American journal of obstetrics and gynecology. 2019;220(4):383.e1–.e17. doi: 10.1016/j.ajog.2018.12.023 30576661

[27] Joseph K. The fetuses-at-risk approach: clarification of semantic and conceptual misapprehension. BMC pregnancy and childbirth. 2008;8:1–3. 18366767 10.1186/1471-2393-8-11 PMC2292135

[28] Healy P, Gordijn SJ, Ganzevoort W, Beune IM, Baschat A, Khalil A, et al. A Core Outcome Set for the prevention and treatment of fetal GROwth restriction: deVeloping Endpoints: the COSGROVE study. American journal of obstetrics and gynecology. 2019;221(4):339.e1–.e10. doi: 10.1016/j.ajog.2019.05.039 31152710

[29] Hoseth E, Joergensen A, Ebbesen F, Moeller M. Blood glucose levels in a population of healthy, breast fed, term infants of appropriate size for gestational age. Archives of Disease in Childhood-Fetal and Neonatal Edition. 2000;83(2):F117–F9. doi: 10.1136/fn.83.2.f117 10952705 PMC1721132

[30] Esakoff TF, Cheng YW, Sparks TN, Caughey AB. The association between birthweight 4000 g or greater and perinatal outcomes in patients with and without gestational diabetes mellitus. American journal of obstetrics and gynecology. 2009;200(6):672.e1–.e4. doi: 10.1016/j.ajog.2009.02.035 19376489

[31] Ju H, Chadha Y, Donovan T, O’ROURKE P. Fetal macrosomia and pregnancy outcomes. Australian and New Zealand Journal of Obstetrics and Gynaecology. 2009;49(5):504–9. doi: 10.1111/j.1479-828X.2009.01052.x 19780734

[32] Stallmach T, Hebisch G, Meier K, Dudenhausen JW, Vogel M. Rescue by birth: defective placental maturation and late fetal mortality. Obstetrics & Gynecology. 2001;97(4):505–9. doi: 10.1016/s0029-7844(00)01208-4 11275018

[33] Alfirevic Z, Devane D, Gyte GM. Continuous cardiotocography (CTG) as a form of electronic fetal monitoring (EFM) for fetal assessment during labour. Cochrane database of systematic reviews. 2013(5). doi: 10.1002/14651858.CD006066.pub2 23728657

[34] Pathak S, Lees CC, Hackett G, Jessop F, Sebire NJ. Frequency and clinical significance of placental histological lesions in an unselected population at or near term. Virchows Archiv. 2011;459(6):565–72. doi: 10.1007/s00428-011-1157-z 22038509

[35] NHS Maternity Statistics, England -2019-20. 2020.

[36] Ruiter L, Ravelli AC, De Graaf IM, Mol BWJ, Pajkrt E. Incidence and recurrence rate of placental abruption: a longitudinal linked national cohort study in the Netherlands. American journal of obstetrics and gynecology. 2015;213(4):573.e1–.e8. doi: 10.1016/j.ajog.2015.06.019 26071916

[37] Motomura K, Ganchimeg T, Nagata C, Ota E, Vogel JP, Betran AP, et al. Incidence and outcomes of uterine rupture among women with prior caesarean section: WHO Multicountry Survey on Maternal and Newborn Health. Scientific reports. 2017;7(1):1–9. 28281576 10.1038/srep44093 PMC5345021

[38] Serena C, Marchetti G, Rambaldi MP, Ottanelli S, Di Tommaso M, Avagliano L, et al. Stillbirth and fetal growth restriction. Journal of Maternal-Fetal and Neonatal Medicine. 2013;26(1):16–20. doi: 10.3109/14767058.2012.718389 22882114

[39] Froen JF, Gardosi JO, Thurmann A, Francis A, Stray-Pedersen B. Restricted fetal growth in sudden intrauterine unexplained death. Acta Obstet Gynecol Scand. 2004;83(9):801–7. doi: 10.1111/j.0001-6349.2004.00602.x 15315590

