# Correction: Perinatal mortality rate and adverse perinatal outcomes presumably attributable to placental dysfunction in (near) term gestation: A nationwide 5-year cohort study

**DOI:** 10.1371/journal.pone.0306376

**Published:** 2024-06-27

**Authors:** Stefanie Elisabeth Damhuis, Hester Dorien Kamphof, Anita C. J. Ravelli Sanne Jehanne Gordijn, Wessel Ganzevoort

The fifth author’s name is incorrectly written. The correct name is: Wessel Ganzevoort. The correct citation is: Damhuis SE, Kamphof HD, Ravelli ACJ, Gordijn SJ, Ganzevoort W (2023) Perinatal mortality rate and adverse perinatal outcomes presumably attributable to placental dysfunction in (near) term gestation: A nationwide 5-year cohort study. PLoS ONE 18(5): e0285096. https://doi.org/10.1371/journal.pone.0285096.
