# Supplementary material for: Perinatal mortality rate and adverse perinatal outcomes presumably attributable to placental dysfunction in (near) term gestation: A nationwide 5-year cohort study
Source: PLoS One. 2023 May 4;18(5):e0285096. doi: 10.1371/journal.pone.0285096 (PMC10159202; doi:10.1371/journal.pone.0285096)
Supplement: S1 File — (DOCX) [file pone.0285096.s001.docx]

APPENDIX

**Table S1** Average weekly prospective antenatal death risk per 1,000 pregnancies according to each different birth weight centile group at 36, 37, 38, 39, 40 and 41 weeks of gestation in the Netherlands from 2015 to 2019.

| Birth weight centile groups | Antenatal death risk per 1,000 pregnancies (fetus at risk) | | | | | |
| --- | --- | --- | --- | --- | --- | --- |
|  | 36 weeks | 37 weeks | 38 weeks | 39 weeks | 40 weeks | 41 weeks |
| p0-p5 | 0.9 | 0.6 | 0.9 | 1.5 | 1.9 | 8.4 |
| p6-p10 | 0.4 | 0.3 | 0.3 | 0.7 | 1.1 | 1.0 |
| p11-p30 | 0.2 | 0.2 | 0.4 | 0.3 | 0.6 | 1.2 |
| p31-p50 | 0.2 | 0.2 | 0.2 | 0.3 | 0.3 | 1.1 |
| p51-p70 | 0.1 | 0.1 | 0.1 | 0.2 | 0.4 | 0.8 |
| p71-p90 | 0.0 | 0.1 | 0.1 | 0.2 | 0.3 | 1.0 |
| p91-p95 | 0.1 | 0.1 | 0.2 | 0.3 | 0.4 | 0.3 |
| p96-p100 | 0.2 | 0.2 | 0.3 | 0.1 | 0.4 | 1.8 |

**Table S2** Perinatal hypoxia related events according to birth weight centile groups for neonates born between 36^+0^ and 41^+6^ weeks’ gestation in the Netherlands from 2015 to 2019.

| Birth weight centile groups | Total pregnancies | Outcome 1  *Adverse pregnancy outcome* | | Outcome 2  *Adverse labor outcome* | | Outcome 3  *Adverse neonatal outcome* | |
| --- | --- | --- | --- | --- | --- | --- | --- |
|  | n | n | % | n | % | n | % |
| p0-p5 | 35,574 | 243 | 0.7% | 5,057 | 14.2% | 1,678 | 4.7% |
| p6-p10 | 34,698 | 122 | 0.4% | 3,384 | 9.8% | 532 | 1.5% |
| p11-p30 | 138,136 | 334 | 0.2% | 10,277 | 7.4% | 888 | 0.6% |
| p31-p50 | 137,535 | 264 | 0.2% | 8,594 | 6.3% | 698 | 0.5% |
| p51-p70 | 135,739 | 231 | 0.2% | 7,499 | 5.5% | 699 | 0.5% |
| p71-p90 | 135,145 | 185 | 0.1% | 6,643 | 4.9% | 900 | 0.7% |
| p91-p95 | 33,842 | 52 | 0.2% | 1,618 | 4.8% | 439 | 1.3% |
| p96-p100 | 33,211 | 72 | 0.2% | 1,712 | 5.2% | 1,108 | 3.3% |
| *Missing* | *1,058* | *9* | *0.9%* | *32* | *3.0%* | *2* | *0.2%* |
| **Total** | **684,938** | **1512** | **0.2%** | **44,816** | **6.5%** | **6,944** | **1.0%** |

Outcome 1 – Severe adverse outcomes of pregnancy: perinatal mortality or HIE (including antenatal death, perinatal death and neonatal death within 28 days).

Outcome 2 – Adverse labor outcomes: Apgar <7 and/or NICU admission (>24 hrs) and/or emergency delivery for fetal compromise.

Outcome 3 – Adverse neonatal outcomes including any of the following: necrotizing enterocolitis, neonatal hypoglycemia, neonatal hypothermia, respiratory distress syndrome, bronchopulmonary dysplasia, and neonatal convulsions.
